# “Continu‐A‐Mente”: continuity of care between hospital and community for people living withdementia and their caregiver with Tailored Activity Program

**DOI:** 10.1002/alz.092496

**Published:** 2025-01-09

**Authors:** Christian Pozzi, Andrea Staglianò, Chiara De Ponti, Claudia Ballabio, Federica Bartoli, Laura Antolini, Maria Cristina Ferrara, Alessandro Morandi, Stefano Cavalli, Laura N. Gitlin, Giuseppe Bellelli

**Affiliations:** ^1^ SUPSI Centre of Competence on Ageing, Manno, Canton Ticino Switzerland; ^2^ San Gerardo dei Tintori IRCCS Foundation, Monza, Lombardia Region Italy; ^3^ Coop. La Meridiana, Monza, Lombardia Region Italy; ^4^ Milano Bicocca University, Milan, Lombardia Region Italy; ^5^ Azienda Speciale Cremona Solidale, Cremona, Lombardia Region Italy; ^6^ Drexel University, Philadelphia, PA USA

## Abstract

**Background:**

Nonpharmacological approaches have been identified as first line treatments for the behavioral and psychological symptoms (NPS) of persons living with dementia (PLWD).

**Methods:**

This is a single‐arm study to evaluate the feasibility of the TAP approach to promote continuity of care between hospital (S. Gerardo hospital ‐ Italy) and community. Inclusion criteria were presence of NPS; autonomy in activities of daily living (Katz’s index ≥ 2); presence of caregiver. On recruitment, PLWD underwent a multidimensional assessment, Clinical Frailty Scale (CFS), Neuropsychiatric Inventory (NPI), Katz’s Index, Timed‐Up‐and‐Go‐test (TUG), number of drugs taken by the patient and caregiver’s sense of competence (SCQ questionnaire). After recruitment, the dyad PwD+caregiver received 8 TAP sessions performed by two occupational therapists, at hospital (two sessions) and at home (6 sessions). Follow‐up was set after 4 mouths. The attrition rate (% of dropouts) was the primary outcome.

**Results:**

51 patients identified as potentially eligible, 21 declined to participate in the project. Currently, 30 dyads have been recruited, with 16 having completed the follow‐up. Overall, there were 6 (20%) dropouts. The mean patient’s age was 81 ±4.8 years. The median CFS patient’s score was 6 and half of patients were taking more than 7 medications per day. Wilcoxon signed‐rank test was conducted on the 16 dyads. From baseline to follow‐up (median, IQR 75%‐25%), there was a significant reduction in the NPI total score (NPI baseline = 17 (10.7‐33.2) NPI follow‐up 15.7 (8‐2 – 24.0), p = .002), caregiver distress (NPI caregiver distress baseline = 9.5 (6.0‐17.0); NPI caregiver distress follow‐up 6.5 (2.2‐11.5); p = .003) and TUG score (TUG baseline = 18.0 (13.0 – 35.5); TUG follow‐up 13.4 (10.5‐21.5); p = .007). Moreover, there was a significant decrease in the SCQ scale (SCQ baseline = 49.0 (44.0‐64.5) SCQ follow‐up 43.0 (36.5‐56.5); p = .001). No patient experiences hospital admissions at follow‐up while 3 patients had accessed emergency department.

**Conclusions:**

Preliminary data are very promising and in line with what was hypothesized in the clinical trial design phase. TAP may be a valuable aid in monitoring the BPSDs of PLWD and avoiding re‐hospitalizations.

